# Dentigerous Cyst of Inflammatory Origin

**DOI:** 10.5005/jp-journals-10005-1076i

**Published:** 2010-09-15

**Authors:** Raghavendra M Shetty, Uma Dixit

**Affiliations:** 1 Associate Professor, Department of Pediatric and Preventive Dentistry, Chhattisgarh Dental College and Research Institute Rajnandgaon, Chhattisgarh, India; 2 Professor, Department of Pediatric and Preventive Dentistry, Dr DY Patil Dental College and Hospital, Nerul, Navi Mumbai Maharashtra, India

**Keywords:** Dentigerous cyst, Enucleation, Inflammatory origin.

## Abstract

A dentigerous cyst encloses a crown of an unerupted tooth by its follicle and is attached to the neck of the tooth. They may be of developmental or inflammatory origin. Dentigerous cyst of inflammatory origin occurs in immature tooth as a result of inflammation from preceding non-vital deciduous tooth or from other source spreading to involve the tooth follicle. These are diagnosed in the first and early part of second decade either on routine radiographic examination or when patient complains of swelling and pain. They generally involve mandibular premolars.

This article presents a case report of an 11-year-old male patient with a dentigerous cyst of inflammatory origin in the left mandibular premolar region with radiographic feature of a large unilocular radiolucency involving unerupted second premolar. The treatment rendered in this patient comprised of surgical enucleation of cyst under local anesthesia along with extraction of mandibular first and second primary molars.

## INTRODUCTION

A dentigerous cyst encloses the crown of an unerupted tooth by expansion of its follicle and is attached to the neck of the tooth.^1^ It is caused by alteration of reduced enamel epithelium after the completion of amelogenesis, which results in fluid accumulation between epithelium and tooth crown.^2,3^

The incidence of dentigerous cyst has been reported as 1.44 in every 100 unerupted teeth.^1^ It is reported to be present more in males than in females and reported to be more commonly associated with unerupted third molars, first and second premolars and canines.^4^

Dentigerous cyst may remain symptom less and may be diagnosed on routine radiographs or patients may give history of slowly enlarging swelling. Pain may be present only when they are secondarily infected.

## CASE REPORT

An 11-year-old male patient reported with a complaint of swelling on the lower left side of the face which was present since one month. The patient gave history of pain in that region since one week on mastication.

Extraoral examination revealed swelling in the left mandibular region which extended laterally 2 cm away from the corner of the mouth to 2 cm prior to the angle of the mandible and inferiorly to the lower border of the mandible. The swelling was firm in consistency and tender on palpation.

**Fig. 1:**
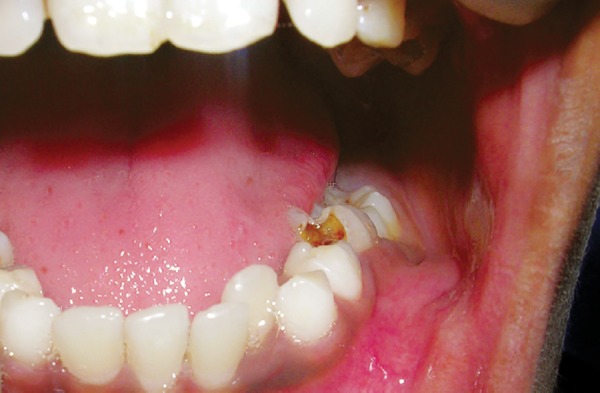
Intraoral view of the patient showing extensive hard swelling

Intraoral examination revealed a swelling extending laterally from distal surface of the mandibular left permanent canine to the distal surface of the mandibular left first permanent molar, superiorly up till the gingival margin and inferiorly obliterating the vestibule. Mandibular left primary first and second molars showed deep carious lesions (Fig. 1). On percussion slight tenderness was present with the second primary molar and grade II mobility was seen in relation to both the primary molars.

**Fig. 2:**
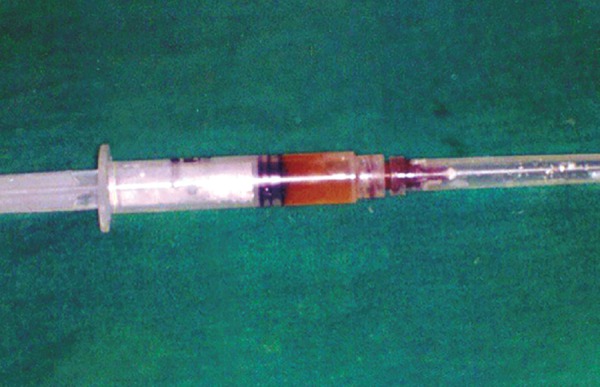
Straw colored fluid aspirated from the swelling

**Fig. 3:**
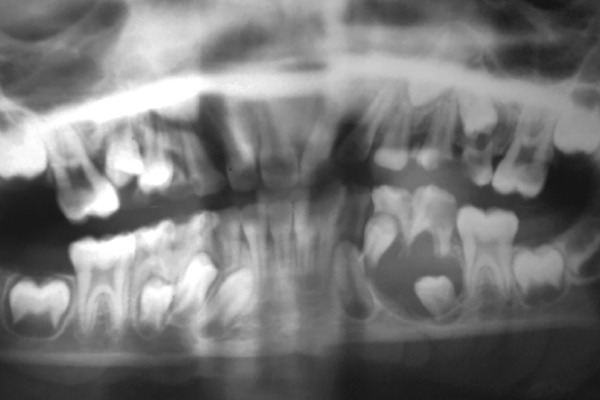
Panoramic radiograph showing a unilocular radiolucency enveloping the unerupted left mandibular second premolar

The panoramic radiograph showed a coronal radiolucency involving pulp with the mandibular left second primary molar. A large unilocular, well-circumscribed radiolucency enveloping the unerupted mandibular left second premolar, attachment being at the cervical margin of the premolar was also seen. Radiograph also showed furcation radiolucency along with external root resorption of the second molar and apical displacement of the unerupted second premolar (Fig. 2). Aspiration of the swelling with a fine needle revealed a straw colored fluid (Fig. 3).

With above findings, it was preliminarily diagnosed as dentigerous cyst with a differential diagnosis of radicular cyst and odontogenic keratocyst. The two treatment options considered were marsupialization of the cyst into the oral cavity or enucleation of cyst.

Treatment procedure was started with administration of local anesthesia by giving an inferior alveolar nerve block on the left side. Sulcular and relieving incisions were given using a number 15 BP blade. Mucoperiosteal flap was raised by periosteal elevator and the cystic lesion with primary molar roots was exposed.

**Fig. 4:**
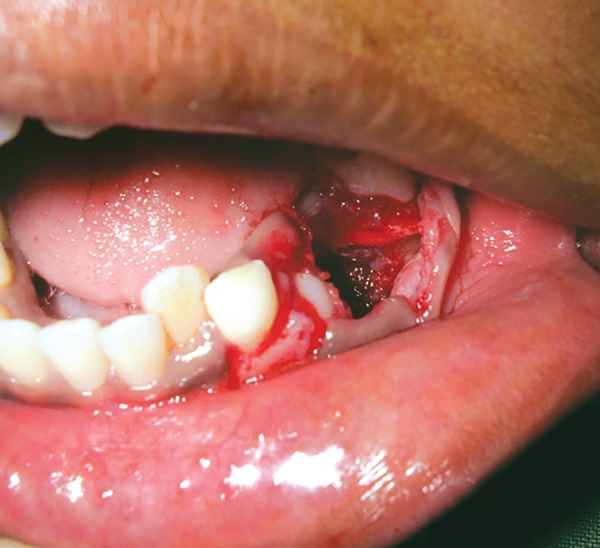
Enucleation of the cyst along with removal of second premolar and extraction of first and second primary molars

**Fig. 5:**
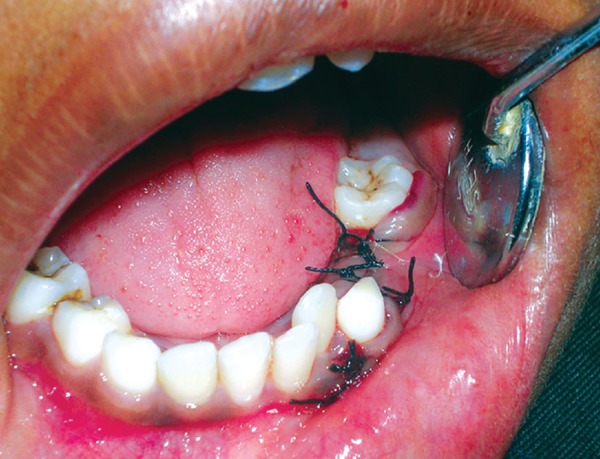
Sutured site after enucleation

It was decided to surgically enucleate the cyst because of the extensive size of the lesion. This was done along with extraction of mandibular left primary first and second molars (Fig. 4). The mucoperiosteal flap was replaced and sutured using 3.0 silk suture materials (Fig. 5).

Macroscopically the specimen measured 3 × 2.5 cm, which was attached to the neck of the left mandibular second premolar and the apical view showed the apical displacement of the tooth bud (Figs 6A and B). Microscopically, hematoxylin and eosin stained section showed few areas of thin, non-keratinized stratified squamous epithelium (2 to 3 layers thick). In certain areas proliferative stratified squamous epithelium was seen. The underlying connective tissue showed moderate infiltration of chronic inflammatory cells mainly composed of lymphocytes and plasma cells with budding capillaries. There was no evidence of malignancy seen (Fig. 7).

On the basis of clinical, radiographic and histo-pathological findings, the present case was diagnosed as dentigerous cyst of inflammatory origin involving the unerupted left mandibular second premolar.

## DISCUSSION

Dentigerous cysts are benign odontogenic cysts associated with crowns of unerupted permanent teeth.^5^ The dentigerous cysts are mostly discovered by routine radiographic examinations or by enlargement of affected region in the jaw.

The pathogenesis of dentigerous cyst is still controversial. Three feasible mechanisms have been proposed for histogenesis of the cyst by Benn and Altini:^2^ They proposed that developmental dentigerous cyst might form a dental follicle and might become secondarily inflamd, a source of inflammation being a non-vital tooth. The second mechanism they proposed was formation of a radicular cyst at an apex of a non-vital deciduous tooth followed by eruption of its permanent sucessor into the radicular cyst resulting in a dentigerous cyst of extrafollicular origin.

They also suggested that follicle of permanent successor might get secondarily infected from either periapical inflammation of a non-vital predecessor or other source leading to a dentigerous cyst formation. Earlier reports of dentigerous cysts associated with non-vital predecessors support this mechanism.^6^ Available evidence in our case indicated that infection of the predecessor (second primary molar) could have been the source of inflammation of the dentigerous cyst.

Unerupted tooth enveloped by a dentigerous cyst may or may not show enamel hypoplasia depending on the time of commencement of a dentigerous cyst. Enamel hypoplasia is seen when a dentigerous cyst commences at an early stage of development of the involved tooth whereas in cases where the cyst originating after the completion of tooth development, enamel hypoplasia is not a significant factor.^7^ In the case presented here, the unerupted premolar did not show enamel hypoplasia and it may be inferred that the cyst may have developed after the crown was fully formed.

**Fig. 6A:**
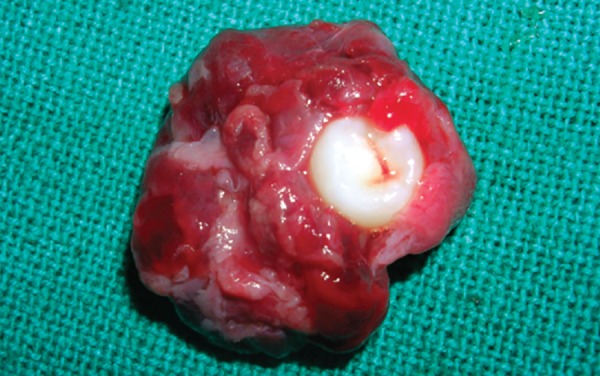
Enucleated cystic lesion along with the second premolar (occlusal view)

**Fig. 6B:**
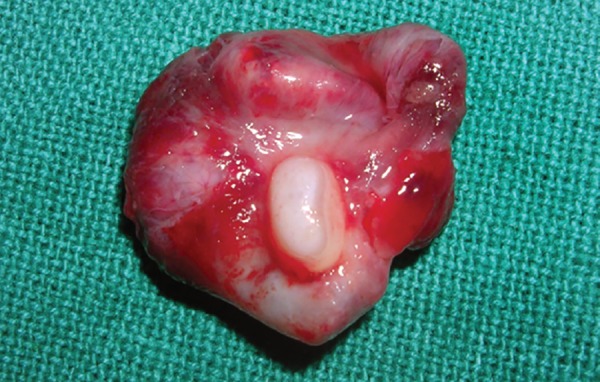
Enucleated cystic lesion along with the apically displaced second premolar (apical view)

**Fig. 7:**
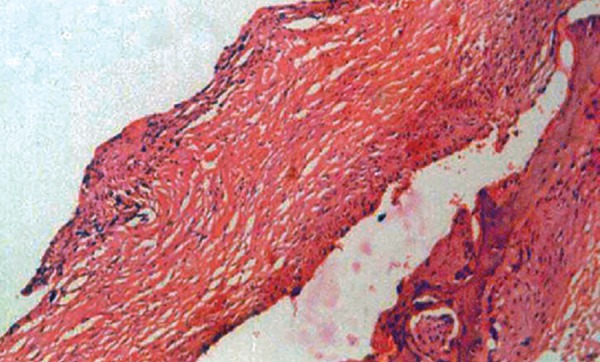
Microscopic section (HE stained) of the cystic lining showing infiltration of inflammatory cells

Dentigerous cysts are usually single lesions as presented in this case. Bilatral and multiple cysts have been reported in patients with syndromes, such as basal cell nevus syndrome, mucopolysaccharidosis and cleidocranial dysplasia.^8-11^

Recommended treatment for dentigerous cyst is marsupialization, if there appears to be a reasonable prospect that the involved tooth might be brought into its normal position in the arch,^12^ as dentigerous cysts are known to recur rarely.^13^ Enucleation of a dentigerous cyst along with removal of the associated permanent tooth is recommended if it shows arrested development, is extensively displaced,^14^ or if the lesion is extensive.

In the case presented here, enucleation of the cyst along with the involved tooth was preferred because of the extensive size of the lesion and apical displacement of the unerupted second premolar.
